# Anatomy of Viral Persistence

**DOI:** 10.1371/journal.ppat.1000523

**Published:** 2009-07-31

**Authors:** Michael B. A. Oldstone

**Affiliations:** Viral Immunobiology Laboratory, Department of Immunology and Microbial Science, The Scripps Research Institute, La Jolla, California, United States of America; University of California San Francisco, United States of America

## Ingredients of a Chronic Viral Infection

The many millions of humans who have life-long virus infections represent a major health issue for the 21st century but also a unique opportunity for investigative virologists. For persistent virus infections to endure, two ingredients are essential. The first is a unique strategy of viral replication; that is, instead of killing its host cell, the pathogen causes little to no damage so it can continue to reside in those cells. The second requirement for persistent virus infection is an immune response that does not react to or remove virus-infected cells. Overall, our knowledge of how viral genes and cellular factors interact to allow persistence to occur is incomplete. Although our libraries contain volumes of facts on this subject, many physiologic functions and interrelationships of viral genes with host genes that establish persistence remain, in large part, unknown. We do know that acutely infected cells express viral peptides, which, when attached to host major histocompatibility complex (MHC) molecules on their surfaces, signal the immune system to kill such cells. However, viruses apply numerous avoidance strategies to persist. One is direct selective pressure to suppress the infected host's innate and/or adoptive immune system that would otherwise destroy them (reviewed [1],[2]) [3]. For example, viruses can alter or interfere with the processing of viral peptides by professional antigen-presenting cells, thereby restricting expression of MHC/peptide complexes on cell surfaces, a requirement for activation and expansion of the T cells that normally remove infected cells. Additionally, viruses can downregulate co-stimulatory and/or MHC molecules also required for T cell signaling and expansion; they can inhibit the differentiation of antigen-presenting conventional dendritic cells (cDCs), and can infect effector T and B cells directly. Similarly, to persist in infected cells, viruses can disrupt the processing or migration of viral peptides or viral peptide/MHC complexes to the cells' surface, thereby removing the recognition signals for activated killer T cells. Finally, viruses that persist frequently infect neurons, which have defects in TAP, a molecule required for the translocation of viral peptides to endoplasmic reticulum (ER) [4],[5]. Perhaps neurons can also actively prevent cytotoxic T lymphocytes (CTLs) or natural killer (NK) cells from degranulating and thereby limit the activity of such virus-removing effector cells. Since neurons are essential to health but rarely regenerate when destroyed, Darwinian selection likely caused them to evolve mechanisms to avoid immunologic assault. Such events would allow infected neurons to escape immune recognition and live, as well as allow viruses to persist in a neuronal safe house.

## Immunological Tolerance as a Mechanism to Explain Viral Persistence

Viruses can cause persistent infection early in life directly from mother to child in utero or in newborns whose immune system is immature, and even later in adults after the immune system has matured. Infection in early life was initially attributed to immunologic tolerance, that is, deletion or removal of cell clones that generate an antiviral immune response [6]. The model for this concept was congenital lymphocytic choriomeningitis virus (LCMV) infection of mice, a life-long symptomless viral carrier state induced by in utero or neonatal infection [7]. Like early hepatitis B or C virus (HBV/HCV) infection of today, LCMV infection studied in the past (LCMV-carrier mouse) was characterized by persistent high titers of virus throughout life without detectable antiviral immune response. This pattern was duplicated in vertically transmitted murine retroviral infections. Thus immunologic tolerance was defined (originally in the LCMV-carrier model) as 1) resistance of normal newborn mice to a viral dose lethal for mature adults; 2) presence of high titers of virus in organs and blood of adults infected in utero or neonatally with resistance to an ordinarily lethal LCMV challenge; and 3) absence of complement-fixing or neutralizing antibodies and of LCMV-specific CTLs (later after the discovery of CTLs) in adults infected in utero or neonatally.

## Problems with the Immunological Tolerance Model

However, results from our work [8],[9] and that of Jamieson and Ahmed [10] indicated major problems with that theory. First, free antiviral antibodies are present not in the circulation but bound to viruses and viral antigens to form v-Ab complexes. These v-Ab complexes deposit primarily in the glomeruli of kidneys, blood vessels, and choroid plexus. Specific anti-LCMV antibodies are readily isolated from the complex by using a low ionic and low pH buffer and quantitatively comprise over 65% of the total immunoglobulin extracted from the glomeruli, a factor 50 to 100 times greater than the specific antiviral LCMV antibody found in the immunoglobulin fraction of adult mice immunized with LCMV [8]. A similar scenario occurs with the in utero or neonatal murine retroviral infections [9]. Further, circulating and glomerular-deposited v-Ab complexes are found in humans with persistent viral infections.

Specific antiviral CTLs are rarely found in adult mice or in humans persistently infected early in life. Reconstitution of virus-specific T cells first shown by adoptive transfer of anti-LCMV-specific CD4 and CD8 T cells readily purged viruses and cleared infection from blood and tissues of LCMV-carrier mice [11]. After virus was cleared, these adult mice, when rechallenged with LCMV, generated a robust specific anti-LCMV CTL response with normal kinetics and intensity and developed immunologic memory, that is, protection against an ordinarily lethal intracerebral challenge with LCMV [10].

Therefore, we conclude that viral infection initiated in neonates or in utero does not lead to deletion of T or B cell clones specific for the virus [8]–[10]. Rather, antibodies are made but do not circulate freely in blood, because they quickly bind to the virus (viral antigen) that is in excess to form immune complexes. Indeed, deposition of such v-Ab complexes leads to tissue injury that accompanies persistent infections. Further, detection of immune complexes is often a reliable marker for persistent virus infection. T cell clones are not deleted, but they are not active or observed until the viral load is lowered or removed. For the treatment of HCV and HBV carriers infected in utero or neonatally, who also fail to show virus-specific T cells, these findings suggest that removal of virus and viral antigens followed by specific immunization may provide a cure.

## Viruses Actively Suppress the Host's Immune Responses

Immune responses reflect the sum of positive versus negative regulators of that response. Negative regulators function to prevent an excessive immunologic response leading to immunopathologic disease. Of those so far identified [12]–[15], IL-10 [13],[14] and PD-1 [12] have claimed the most interest. They evidently function via separate pathways [13],[16], so combination therapy that neutralizes both has been more effective than removal of either one alone [16].

Viruses that initiate persistent infections in juveniles or adults take advantage of this negative regulatory system by actively causing hosts to make regulators that turn off or exhaust the expected antiviral immune response. The discovery that viruses can escape immunologic attack and persist by this means again came from the study of LCMV infection in its natural murine host [12]–[14] and has been extended to studies of humans persistently infected with HIV and HCV [17]–[21] and primates infected with simian immunodeficiency virus [22].

In the LCMV persistent infection induced in adults, IL-10 is produced primarily by virus-infected cDCs and perhaps by B cells [13]. In vivo, neither CD4 nor CD8 T cells produce significant amounts of IL-10. Still not clear, though, is which cells IL-10 affects. What is known is that by 9 days after a persistent LCMV infection is initiated in adult mice, T cells become unresponsive; that is, they fail to or poorly lyse virus-infected targets and do not make the positive immune regulators IL-2, interferon-γ, or TNF-α. However, when IL-10 is blockaded with antibody to IL-10 receptors, T cell functions are restored, and their numbers increase sufficiently to clear virus from blood and tissues [13]. Interestingly, although IL-10 plays a major role in T cell exhaustion of adult mice persistently infected with LCMV, as yet no similar role for IL-10 has been found in the persistent infection of adults infected in utero or neonatally (D. Brooks, D. McGavern, M. Oldstone, unpublished data).

PD-1 expression increases on T cells during persistent LCMV infection and, as stated above, these T cells become hyporesponsive or exhausted and cannot clear infection [12],[15]. Blockade of PD-L1 by specific antibody restores T cell function, which then allows these effector T cells to control the virus infection [12].

The exploration and understanding of negative immune regulators, including IL-10 and PD-1, are still at an early stage; nevertheless, the implications are important and profound. First, exhausted or hyporesponsive T cells found in persistent infections can be resurrected to functional capacity. This is true not only for the LCMV model in which the phenomenon was uncovered [12]–[14], but also for HIV and HCV in vitro and simian immunodeficiency virus in vivo [17]–[22]. Second, the environment in which negative regulators are induced by the virus provides one of the major problems in treating persistent infections. Our prediction is that when vaccination strategies to treat persistent infection fail, the likely cause is not a faulty immunogen or adjuvant approach, but overwhelming control of the environment by negative regulators. Indeed, therapeutic vaccination against ongoing persistent LCMV infection was effective only when IL-10 or PD-L1 was first neutralized [23],[24].

Clearance of a persistent LCMV infection requires virus-specific CD4 T cell help to assist virus-specific CD8 T cells [25]–[28]. Recently, IL-21 was identified as essential for CD4 T cell help and allowing CD8 T cells to control the persistent infection [29]–[31].

## The Future and Potential Applications for Treating Persistent Viral Infections

Currently our laboratory and others are engaged in the discovery of additional negative immune regulators and their signaling pathway(s) using gene chip and forward genetics technology. These projects have a multitude of applications. Some examples are the development of pharmacologic small molecules as effective antagonists of negative immune regulators, the use of transient negative regulator blockers as an adjuvant approach to enhance both prophylactic and therapeutic vaccination, and the determination of how long during the course of persistent virus infection exhausted T cells can be rescued to become antiviral effector T cells. As always, the goal is to understand basic principles in viral pathogenesis and to extend results in the murine model to resolve persistent infections of humans.[Fig ppat-1000523-g001] [Fig ppat-1000523-g002]

**Figure 1 ppat-1000523-g001:**
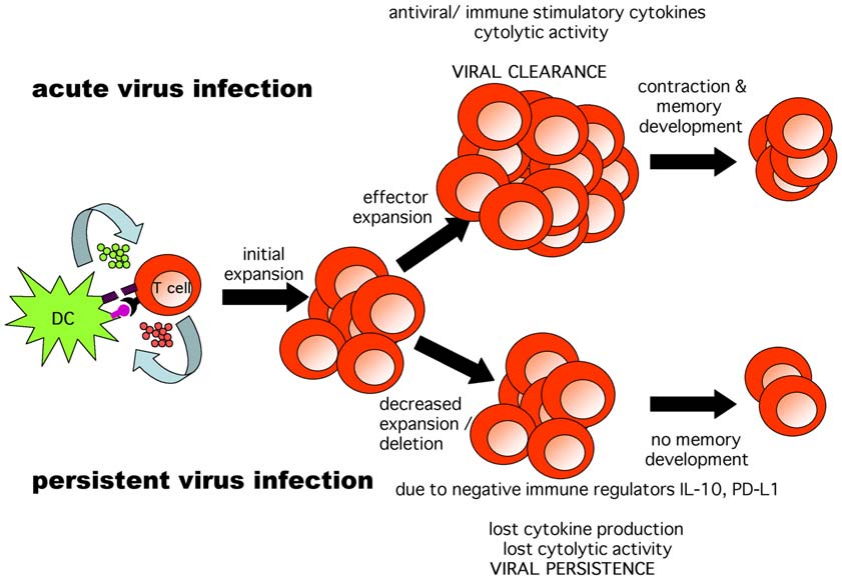
Cartoon of the initiation of an uncontrolled persistent virus infection or a controlled acute virus infection in immunocompetent adults. Dendritic cells (DC) present viral peptide/MHC complexes to activate T cells. There is an initial expansion phase following infections that lead to either clearance of the virus or virus persistence. For virus clearance, following the acute infection positive immune regulators (IL-2, IFN-γ, TNF, etc.) are generated that expand the effector virus-specific T cell pool, resulting in elimination of virally infected cells, termination of the infection, and resultant development of immune memory. By contrast, with viruses that persist there is a decreased expansion, and in some cases deletion, of virus-specific T cells. Remaining T cells become exhausted or hyporesponsive and are defective in the release of positive immune regulators and hence are unable to terminate the virus infection. The cause is the virus' induction of negative regulators of the immune response, i.e., IL-10 and PD-L1, and the cure is the blockade of such negative regulators with appropriate antibodies (see [12]–[14]).

**Figure 2 ppat-1000523-g002:**
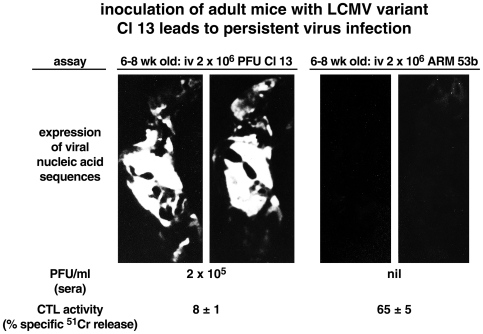
The scenario of virus induction of negative regulators leading to T cell hyporesponsiveness sprung from experimental analysis of LCMV infection in its natural murine host using inoculation of parental LCMV Armstrong strain 53B or its variant, LCMV Cl 13. The 10.7-kb genome of these viruses differs by only six nucleotides that code for three amino acids. One amino acid located in the viral spike protein GP-1 at aa 260 (Leu Cl 13/Phe ARM 53b) is responsible for high affinity binding (2.5 logs higher affinity for Cl 13 over ARM 53b) for the LCMV receptor alpha-dystroglycan, which is located in the immune system, preferentially on DCs (see [32],[33]). A second important mutation is in the viral polymerase at aa 1079 (Leu Cl 13/Gln ARM 53b) and is associated with enhanced transcription and replication of LCMV Cl 13. Recent studies have also implicated infection of the fibroblastic reticular cells in lymphoid organs as contributing to the persistent infection (see [34]). Figure 2 shows this using a whole body section of a mouse. The tissue section was placed on a membrane and stained with a riboprobe to LCMV at 30 days after initiation of LCMV infection with either LCMV Cl 13 or LCMV ARM 53b. The presence of viral nucleic acids in mice receiving Cl 13 correlates directly with high titers of virus carried in the sera (PFU/ml) at 30 days post-infection and the lack of a CTL response observed 7 days after initiation of infection. By comparison, mice receiving LCMV ARM 53b at 30 days post-infection fail to display viral nucleic acid sequences or virus in their sera, as virus has been successfully purged. Further, mice infected with LCMV ARM 53b generate a robust CTL response 7 days following virus infection.
